# Overexpression of Two *PsnAP1* Genes from *Populus simonii* × *P. nigra* Causes Early Flowering in Transgenic Tobacco and *Arabidopsis*

**DOI:** 10.1371/journal.pone.0111725

**Published:** 2014-10-31

**Authors:** Tangchun Zheng, Shuang Li, Lina Zang, Lijuan Dai, Chuanping Yang, Guan-Zheng Qu

**Affiliations:** State Key Laboratory of Tree Genetics and Breeding (Northeast Forestry University), Harbin, China; The National Orchid Conservation Center of China; The Orchid Conservation & Research Center of Shenzhen, China

## Abstract

In *Arabidopsis*, *AP1* is a floral meristem identity gene and plays an important role in floral organ development. In this study, *PsnAP1-1* and *PsnAP1-2* were isolated from the male reproductive buds of poplar (*Populus simonii* × *P. nigra*), which are the orthologs of *AP1* in *Arabidopsis*, by sequence analysis. Northern blot and qRT-PCR analysis showed that *PsnAP1-1* and *PsnAP1-2* exhibited high expression level in early inflorescence development of poplar. Subcellular localization showed the PsnAP1-1 and PsnAP1-2 proteins are localized in the nucleus. Overexpression of *PsnAP1-1* and *PsnAP1-2* in tobacco under the control of a CaMV 35S promoter significantly enhanced early flowering. These transgenic plants also showed much earlier stem initiation and higher rates of photosynthesis than did wild-type tobacco. qRT-PCR analysis further indicated that overexpression of *PsnAP1-1* and *PsnAP1-2* resulted in up-regulation of genes related to flowering, such as *NtMADS4*, *NtMADS5* and *NtMADS11*. Overexpression of *PsnAP1-1* and *PsnAP1-2* in *Arabidopsis* also induced early flowering, but did not complement the *ap1-10* floral morphology to any noticeable extent. This study indicates that *PsnAP1-1* and *PsnAP1-2* play a role in floral transition of poplar.

## Introduction

In *Arabidopsis*, flowering time is controlled by photoperiod, vernalization, gibberellin, autonomous, and endogenous pathways. Signals from different pathways interact to regulate floral development through the action of flowering pathway integrators, such as *FLOWERING LOCUS T* (*FT*) and *SUPPRESSOR OF OVEREXPRESSION OF CO1* (*SOC1*) [1]–[3]. Integrators act upstream of floral meristem identity genes to determine the fate of the shoot apical meristem (SAM) [3]. In *Arabidopsis*, *AP1* is a floral meristem identity gene, and plays an important role in the transition from vegetative to reproductive growth. Transgenic *Arabidopsis* plants which constitutively express the *AP1* gene show transformation of apical and lateral shoots into flowers, and these plants flower much earlier than wild-type plants [4]. Besides being a floral meristem identity gene, *AP1* plays an important role in floral organ development in *Arabidopsis*. In strong *Arabidopsis ap1* mutants, sepals are converted to bract-like structures, petals are absent, and the bract-like organs of the first whorl subtend secondary flowers in the second whorl [5]–[7]. Some *AP1* genes from other plant species were studied. In citrus, ectopic expression of *AtAP1* induced flowering within the first year, and the shortening of the juvenile period was stable [8]. Overexpression of *MdMADS5*, an *APETALA1*-like gene in apple, caused early flowering in transgenic *Arabidopsis* [9]. Overexpression of birch *BpAP1* in tobacco and birch also caused early flowering and dwarfism, giving these plants an obviously shortened juvenile phase [10], [11].

Poplar plays key roles in forest production and ecological projects, and is a model forest tree for biological study. Modification of flowering can play an important role in the breeding of poplars. Several poplar flowering-related genes were studied. The expression pattern of a MADS-box gene (*PdPI*) from *Populus deltoides*, an ortholog of the *Arabidopsis PISTILLATA* (*PI*) gene, was characterized by Zhang et al. [12]. Hsu reported overexpression of *FT2* gene from *P. deltoids*, a relative of the *Arabidopsis* flowering-time gene *FT*, induced flowering in *Populus alba × P. tremula* within 1 year [13]. Research by Hsu also revealed that reproductive onset is determined by *FT1* in response to winter temperatures, whereas vegetative growth and inhibition of bud set are promoted by *FT2* in response to warm temperatures and long days in the growing season [14]. Up to date, studies of the controlling floral initiation in poplar are limited. The function of poplar *AP1* is still not clear. To investigate the functional similarity between poplar *AP1* and their *Arabidopsis thaliana* counterparts, two *AP1* genes were cloned from *Populus simonii* × *P. nigra*, and their expression patterns in poplar and ectopic expression in tobacco and *Arabidopsis* plants were determined in this study.

## Materials and Methods

### Plant material and growth conditions

The *P. simonii* × *P. nigra* cross was obtained from campus of Northeast Forestry University in Heilongjiang Province, China. Various poplar tissues (flower buds, stems, roots, and leaves) were sampled, immediately frozen in liquid nitrogen, and stored at −80°C before the isolation of total RNA. The tobacco (*Nicotiana tabacum* L.) seedlings were grown in pots containing a mixture of turf peat, and sand (2∶1 v/v) and *Arabidopsis* (*Arabidopsis thaliana* Col-0) were grown in pots containing a mixture of turf peat, vermiculite, and sand (3∶1∶1 v/v) in a greenhouse under controlled conditions with 60–75% relative humidity and an average temperature of 22±2°C. Cool white fluorescent lights supplied photons at 200 µmol m^−2^ s^−1^.

### Isolation of PsnAP1

The two *PsnAP1* (*PsnAP1-1*, *PsnAP1-2*) cDNAs for the open reading frame (ORF) of *PsnAP1* were cloned from total RNA of poplar male flower buds using RT-PCR. The primers used to amplify *PsnAP1* were listed in Table S1. The PCR cycle profile was: an initial denaturation of 94°C for 4 min; 35 cycles of 94°C for 30 s, 58°C for 30 s and 72°C for 1 min; a final extension of 72°C for 7 min. The deduced *PsnAP1* proteins were characterized using Expasy tools (http://www.expasy.org/tools). Sequence alignments were performed using the ClustalX program and MEGA version 4.1 [15].

### PsnAP1 gene subcellular localization analysis

The two cDNAs for ORF of *PsnAP1* gene were respectively cloned into the vector pTH2 to generate the *PsnAP1*-*GFP* fusion gene driven by a CaMV 35S promoter as described by Niwa [16]. The primers used to amplify *PsnAP1* were listed in Table S1. The *PsnAP1*-*GFP* constructs were transformed into onion epidermal cells by particle bombardment (Bio-Rad PDS-1000/He System, USA). PsnAP1-GFP fusion protein transient expression were observed using Zeiss Confocal Microscopy.

### Quantitative Real-Time PCR (qRT-PCR) Analysis

To detect expression level of *PsnAP1* in poplar, total RNA was isolated from male flower buds in different months, young leaves, old leaves, stems, and roots. For the qRT-PCR analysis, 1 µg aliquot of total RNA treated with DNase I (Invitrogen, Carlsbad, CA, USA) was reverse-transcribed using PrimeScript RT reagent Kit (TaKaRa, Dalian, China). SYBR Premix EX Taq II (TaKaRa, Dalian, China) and MJ Opticon 2 System (Bio-Rad, Hercules, CA, USA) were used according to the manufacturer’s instructions. The gene-specific primers were used for quantifying the transcripts of *PsnAP1-1* and *PsnAP1-2* with *Actin* from poplar as internal references. To estimate the transcript level of flowering-related genes in transgenic and wild-type tobacco with qRT-PCR, aerial parts of the 25-day-old tobacco plants were harvested when inflorescences were invisible. Specific primers for flowering-related genes were used for qRT-PCR, with *Ntactin* gene as internal reference. All the primers in present study for qRT-PCR were listed in Table S1. Each reaction was conducted in triplicate to ensure reproducibility of results. Expression levels were calculated from the cycle threshold according to the delta-delta CT method [17].

### Construction of plant expression vectors and plant transformation

To express the two *PsnAP1* genes in plants, these genes with different restriction enzymes sites were cloned with the primers listed in Table S1. The PCR product of *PsnAP1-1* was digested with *Xba* I and *Kpn* I, then inserted into pROKII between *Xba* I and *Kpn* I sites, forming pROKII-*PsnAP1-1*. PCR product of *PsnAP1-2* was digested with *Bam*H I and *Sac* I, then inserted into pROKII between *Bam*H I and *Sac* I, to form pROKII-*PsnAP1-2*. The recombinants were identified by PCR and sequencing analysis. At last, the resulting binary vectors, pROKII-*PsnAP1-1* and pROKII-*PsnAP1-2* were respectively transferred into *Agrobacterium tumefaciens* strain EHA105 using the freeze-thaw transformation method [18].

Leaf discs of tobacco were transformed by the *Agrobacterium*-mediated method as follow. Tobacco leaf disks were precultured in the differentiation medium (MS medium +20 g L^−1^ sucrose +0.05 mg L^−1^ 1-Naphthylacetic acid, NAA (Sigma, USA) +0.5 mg L^−1^ N6-Benzyladenine, 6-BA (Sigma, USA) +7 g L^−1^ agar) for 2 d, then inoculated with *Agrobacterium* suspension for 3–5 min. Leaf disks were co-cultivated for 2 d in differentiation medium without antibiotics, then transferred to MS differentiation selection medium containing 40 mg L^−1^ Kanamycin (Km) and 500 mg L^−1^ Cefotaxime Sodium (Cef) at 25°C in a 16 h light/8 h dark photoperiod at an intensity of ∼2000 lux. After 15 d of culture, the explants with putative transgenic shoot buds were transferred to selection medium with 50 mg/L Km and 500 mg L^−1^ Cef.

To study gene function, *Arabidopsis* wild-type and *ap1-10* mutant (*A*. *thaliana* Col-0, Stock: CS6230) were transformed with pROKII-*PsnAP1-1* and pROKII-*PsnAP1-2* constructs to overexpress these *PsnAP1* genes. *Arabidopsis* flowers were dipped with *A. tumefaciens* EHA105 suspended in a solution containing 10 mM MgCl_2_, 5% sucrose and 0.02% Silwet L-77, and then the plants were covered with a plastic bag and incubated in a growth chamber at 23°C for 1 d and finally allowed to grow in a growth chamber as usual. Finally, the seeds were harvested and selected on selection medium containing 50 mg L^−1^ Km.

### Northern blot analysis

For Northern analysis, 10 µg of total RNA of poplar, tobacco or *Arabidopsis* was separated on 1% agarose denaturing formaldehyde gel and transferred to a Hybond-N^+^ nylon membrane and fixed with UV cross-linking (254 nm, 8 min). Hybridization and detection were conducted in accordance with the manufacturer’s instructions (DIG Northern starter Kit, Roche).

### Histological microscope observations

For histological observation of vegetative apex of tobacco, 25-day-old seedlings were collected and fixed by immersion in a formalin:acetic acid:ethanol:water solution (FAA, 1∶1∶9∶9 v:v) at room temperature for 48 h and then stored in 70% ethanol. The fixed material was then stained with hematoxylin solution (Ehrlich) for 4 d. After dehydration through an ethanol series (30, 50, 70, 85, 95, and 100%; 2 h at each concentration), the samples were embedded in paraffin wax (58–60°C). Semi-sections (8 µm) were made from longitudinal-sectional slices of using a microtome (HM 340 E; Microm International GmbH, Walldorf, Germany) and fixed on glass slides. The sections were de-waxed in xylene for 5–10 min and observed under a light microscope (Docuval, Carl Zeiss, Germany).

For histological observation of tobacco stems, 25-day-old seedlings were collected and fixed in FAA solution. Transverse sections of 10 mm thick were cut as described above. After deparaffinization and rehydration, sections were stained with 1% solution of safranin T (Sigma, USA) for 24 h and then with 0.1% solution of toluidine blue (Sigma, USA) for 30 s. These sections were dehydrated in a graded ethanol series, mounted on glass slides, fixed in resin, covered with coverslips, and observed under a light microscope described above.

### Histochemical staining for starch

For starch-iodine staining, the seedlings were treated under dark for 24 h, then the young leaves of 30-day-old tobacco under light treatment for 3 h were harvested and fixed in FAA solution for 24 h. After chlorophyll extraction, starch was stained with iodine solution for 20 min, then washed with plenty of deionized water. The result was obtained by the digital camera.

### Chlorophyll content analysis

For measuring the content of chloroplast, the third leaf from the top of 30-day-old tobacco was collected. Chlorophyll content (Chl.) of leaves was measured by the DMSO method as described by Barnes et al. [19] and Shinano et al. [20].

## Results

### Isolation and analysis of the PsnAP1-1 and PsnAP1-2

**Figure 1:**
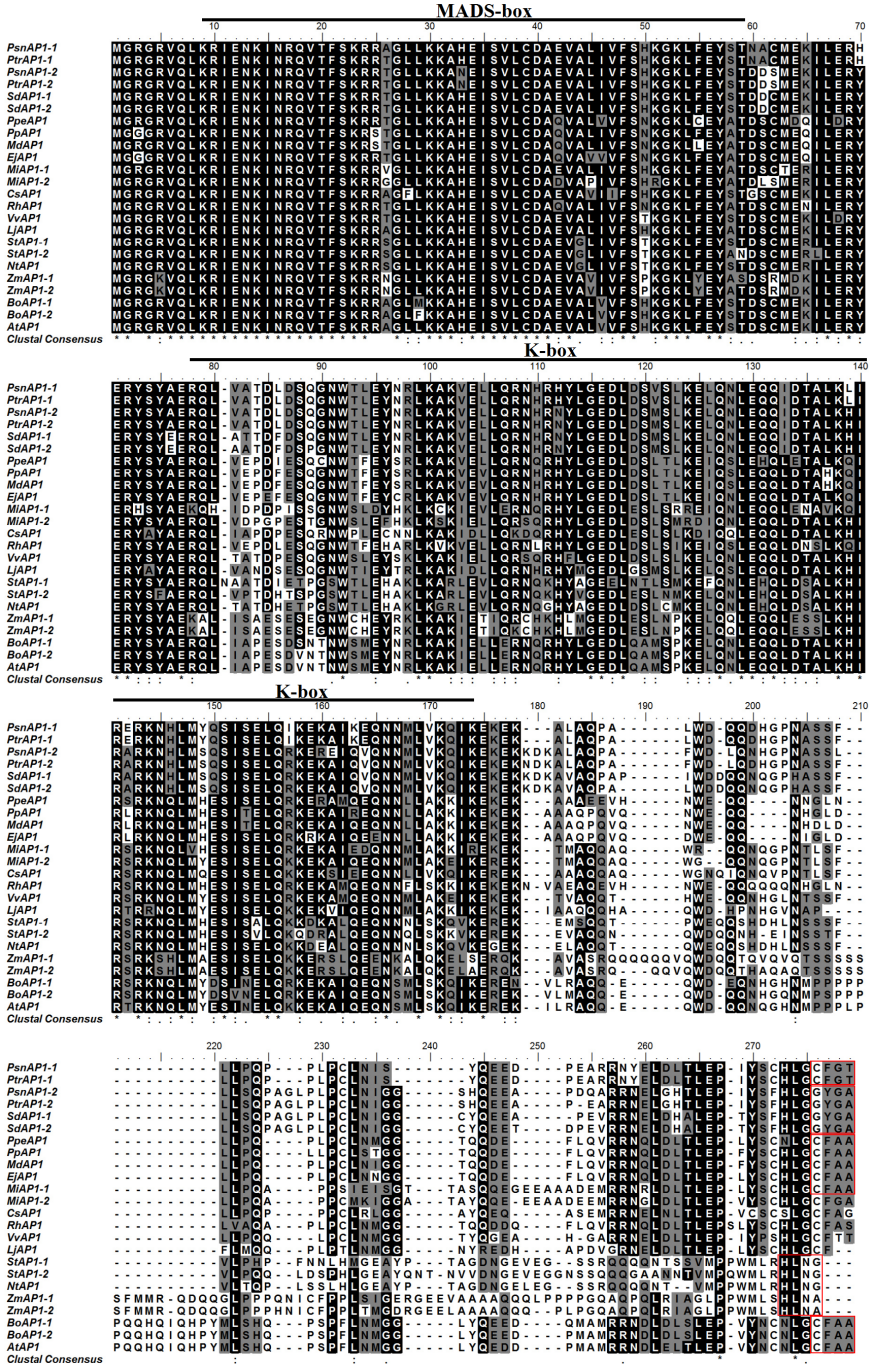
Alignment of the deduced amino acid sequences of AP1 homologs including, PsnAP1-1 and PsnAP1-2. PtrAP1-1 and PtrAP1-2 from *Populus. trichocarpa* (AAT39554, AAT39556), SdAP1-1 and SdAP1-2 from *Salix discolor* (AAY82244, AAY82245), PpeAP1 from *Prunus persica* (ABU63953), PpAP1 from *Pyrus pyrifolia* (ABP93402), MdAP1 from *Malus domestica* (BAH10867), EjAP1 from *Eriobotrya japonica* (AAX14151), MiAP1-1 and MiAP1-2 from *Mangifera indica* (ACL68407, ACL68408), CsAP1 from *Citrus sinensis* (AAR01227), RhAP1 from *Rosa hybrid cultivar* (ACS74806), VvAP1 from *Vitis vinifera* (ACZ26529), LjAP1 from *Lotus japonicus* (AAX13296), StAP1-1 and StAP1-2 from *Solanum tuberosum* (XP_006345101, NP_001275142), NtAP1 from *Nicotiana tabacum* (AAD01421), ZmAP1-1 and ZmAP1-2 from *Zea mays* (DAA59399, DAA42679), BoAP1-1 and BoAP1-2 from *Brassica oleracea* (CAD47853, AAB08875), and AtAP1 from *Arabidopsis thaliana* (NP_177074). Black shadows indicate identical amino acids; dashed lines indicate gaps to optimize the alignment. The same motif in C terminus is in red square.

Two homologous *AP1* cDNAs were cloned from poplar male flower buds of *P. simonii × P. nigra* using RT-PCR, and named *PsnAP1-1* (GenBank No. KC866354) and *PsnAP1-2* (GenBank No. KC866355). *PsnAP1-1* contains a 726 bp open reading frame (ORF) corresponding to a deduced protein of 241 amino acids, and the estimated molecular weight and isoelectric point of the putative protein were 28.1 kD and 8.19. *PsnAP1-2* contains an open reading frame (ORF) of 750 bp, encoding 249 amino acids with a predicted molecular mass of 28.7 kD and a pI of 9.07. The two protein sequences exhibited significant Pfam matches with SRF-type transcription factor (9–59 aa; PF00319) and K-box region (78–174 aa; PF01486) (http://pfam.sanger.ac.uk/), suggesting that these two genes belong to the MADS gene family. PsnAP1-1 shared 82% identity with PsnAP1-2 at the amino acid level. PsnAP1-1 and PsnAP1-2 shared 71% and 67% homology with *A. thaliana* AP1, respectively (Fig. 1), indicating similar functions of these three proteins. The deduced protein sequences of PsnAP1 also shared high sequence homology with the AP1 proteins previously characterized in other plants (Fig. 1). The two *PsnAP1* genes are located in two distinct chromosomes: *PsnAP1-1* gene on chromosome 8 and *PsnAP1-2* gene on chromosome 10, indicating that they are paralogs instead of alleles.

### Subcellular localization of PsnAP1

**Figure 2:**
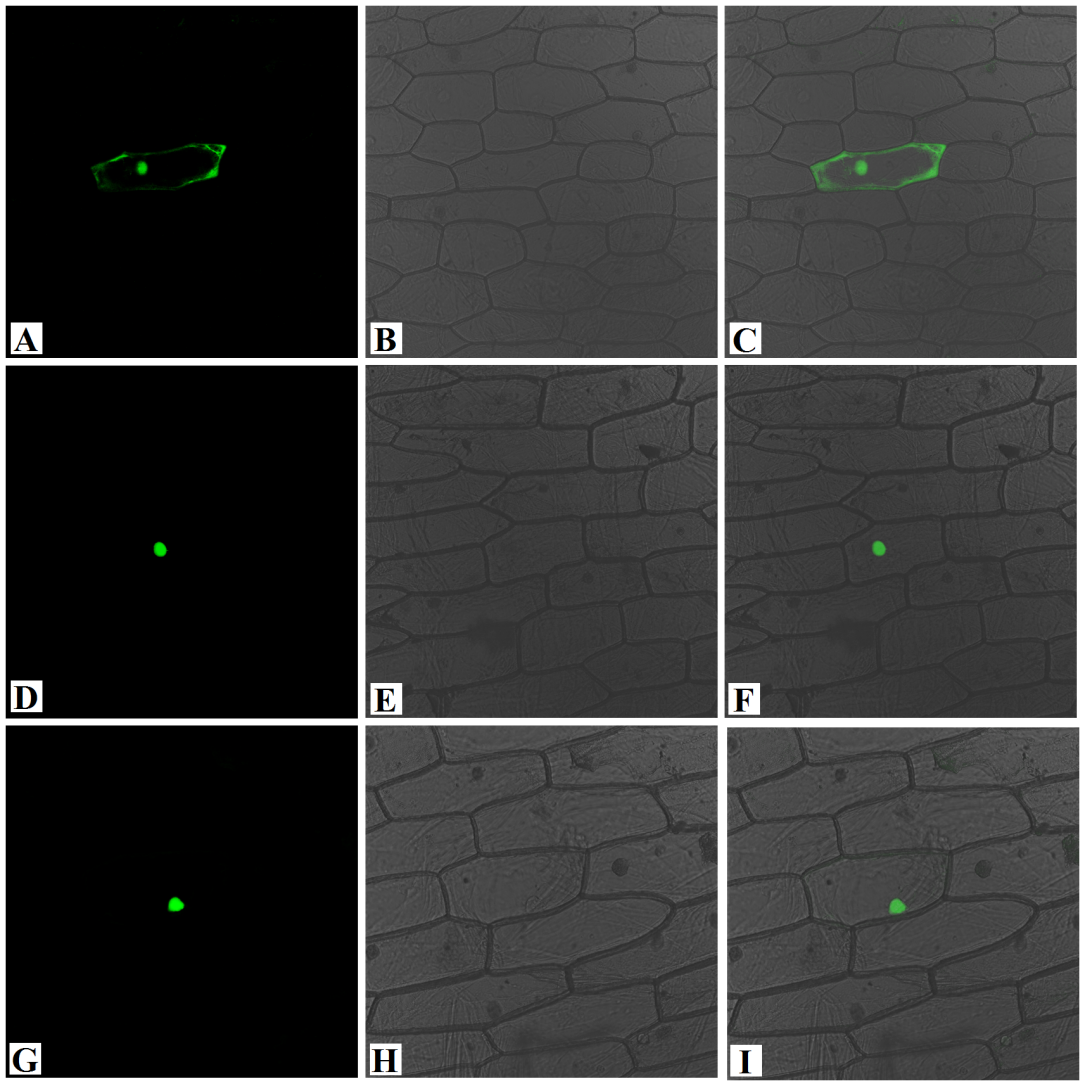
Subcellular localization analysis of the PsnAP1-1 and PsnAP1-2. PsnAP1-1-GFP, PsnAP1-2-GFP fusion, and GFP alone were each expressed transiently under the control of a CaMV 35S promoter in onion epidermal cells and observed under a confocal microscope. The photographs were taken in a dark field (**A**, **D**, **G**), so that green fluorescence could be used to determine localization, in a bright field to examine cell morphology (**B**, **E**, **H**), and in combination (**C**, **F**, **I**). (**A**, **B**, **C**) The cell is transiently expressing the GFP control. (**D**, **E**, **F**) The cell is expressing the PsnAP1-1-GFP fusion protein. (**G**, **H**, **I**) The cell is expressing the PsnAP1-2-GFP fusion protein.

The subcellular localization of the PsnAP1 proteins was examined by introduction of the PsnAP1-GFP fusion protein into onion epidermal cells by particle bombardment. While the control GFP fluorescence signals were both detected in cytoplasm and nucleus (Fig. 2A, B, and C), PsnAP1-1-GFP signals were only observed in the nucleus (Fig. 2D, E, and F). Similarly, PsnAP1-2-GFP fluorescence signals were also only detected in the nucleus (Fig. 2G, H, and I).

### Pattern of expression of PsnAP1 in different tissues

**Figure 3:**
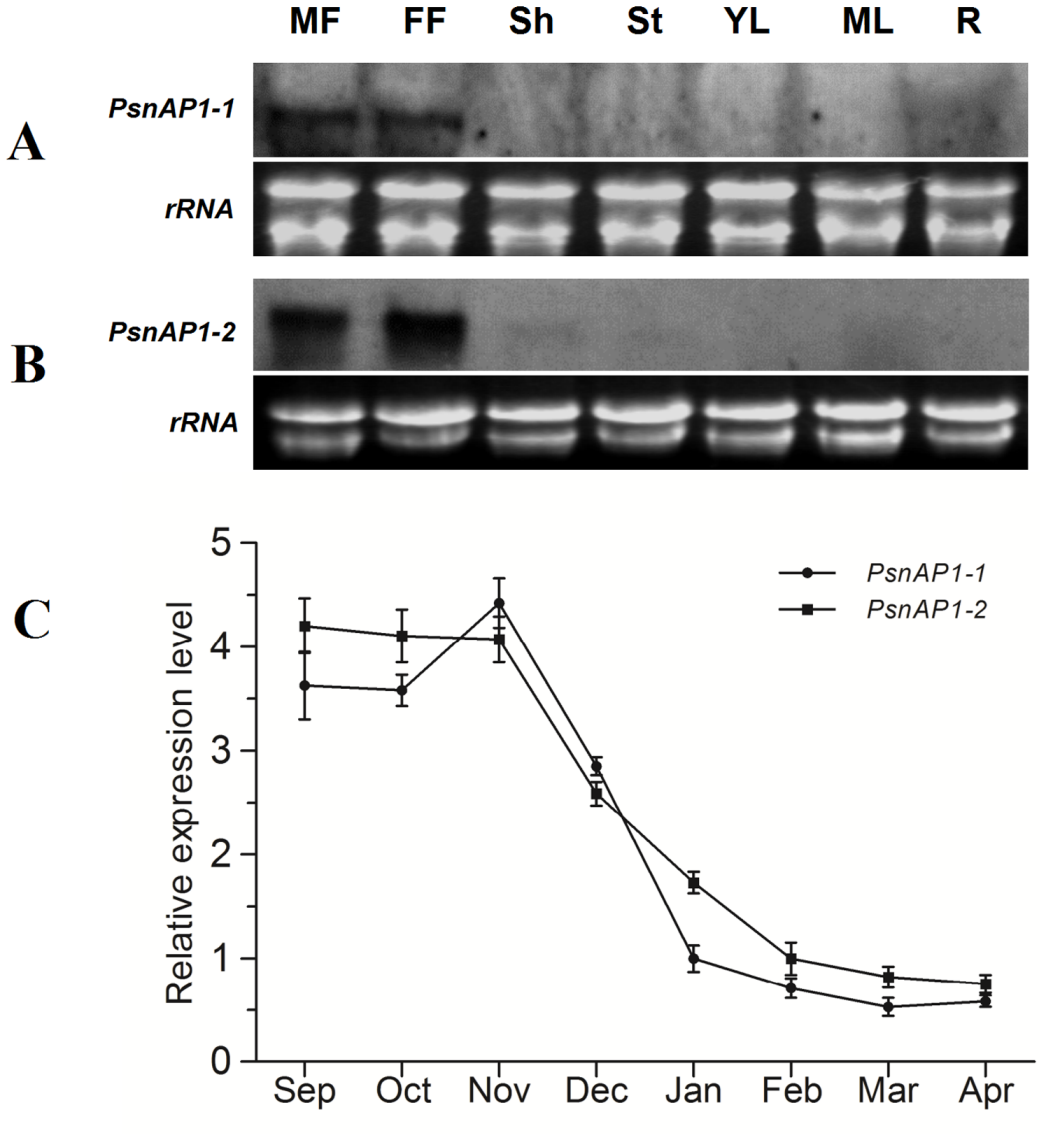
Relative expression levels of *PsnAP1-1* and *PsnAP1-2* in different tissues. (**A**, **B**) *PsnAP1-1* (**A**) and *PsnAP1-2* (**B**) mRNA levels were determined by Northern blot analysis. Total RNA (10 µg) was used. All tissues were collected from *P. simonii* × *P. nigra*. MF male flower, FF female flower, Sh shoots, St stem, YL young leaf, ML mature leaf, R root. (**C**) *PsnAP1-1* and *PsnAP1-2* mRNA levels of male buds in different months were determined by qRT-PCR.

**Figure 4:**
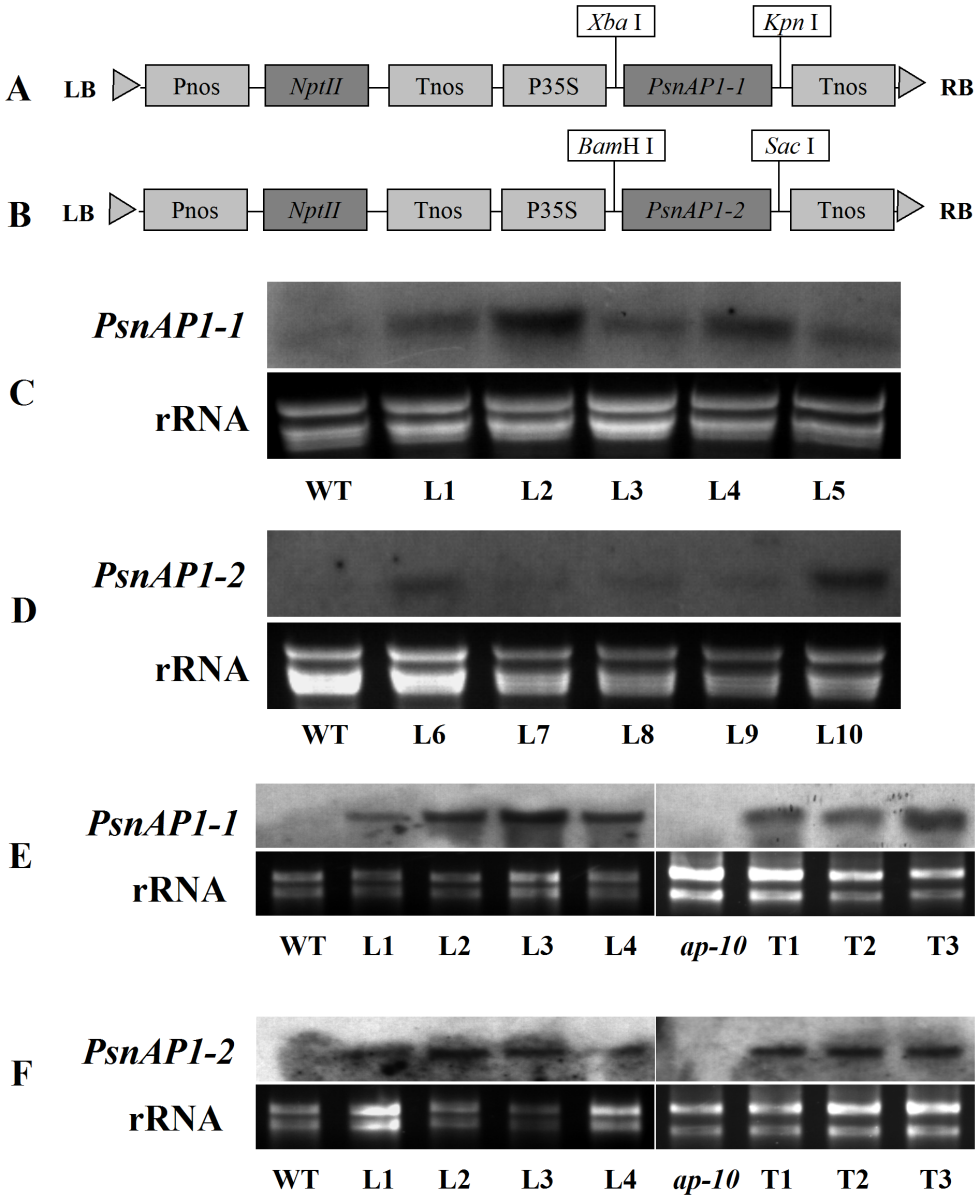
Northern blot analysis of *PsnAP1-1* and *PsnAP1-2* expression in transgenic plants. (**A**, **B**) Schematic of the T-DNA region of the binary vector pROKII-*PsnAP1-1* and pROKII-*PsnAP1-2*. LB left border, Pnos nopalina synthase promoter, *NptII*, kanamycin resistance gene, Tnos nopalina synthase terminator, P35S CaMV 35S promoter, RB right border. (**C**) Northern blot analysis of *35S::PsnAP1-1* in transgenic tobacco. Leaves were sampled from wild-type and transgenic plants. WT wild-type, L1–5 five *35S::PsnAP1-1* lines. (**D**) Northern blot analysis of *35S::PsnAP1-2* in transgenic tobacco. Leaves were sampled from wild-type and transgenic plants. WT wild-type, L6–10 five *35S::PsnAP1-2* lines. (**E**) Northern blot analysis of *35S::PsnAP1-1* in transgenic *Arabidopsis*. Leaves were sampled from wild-type and transgenic plants. WT wild-type, L1–5 five *35S::PsnAP1-1* lines, *ap1-10 ap1-10* mutant, T1–3 three *35S::PsnAP1-1* lines in *ap1-10* mutants. (**F**) Northern blot analysis of *35S::PsnAP1-2* in transgenic *Arabidopsis*. Leaves were sampled from wild-type and transgenic plants. WT wild-type, L1–5 five *35S::PsnAP1-2* lines, *ap1-10 ap1-10* mutant, T1–3 three *35S::PsnAP1-2* lines in *ap1-10* mutants.

Northern blotting and qRT-PCR were used to reveal *PsnAP1-1* and *PsnAP1-2* transcripts in different poplar tissues (Fig. 3). Northern blotting demonstrated that *PsnAP1-1* and *PsnAP1-2* are highly expressed in both male and female inflorescence but are expressed extremely in low levels in shoots, stems, leaves, and roots (Fig. 3A, B). This expression profile was similar to that of the *AP1* gene in birch as reported by Qu et al. [10]. qRT-PCR analysis was used to detect the expression level of *PsnAP1* in developmental male floral buds from September to the following April, and results indicated high transcription levels of *PsnAP1-1* and *PsnAP1-2* from September to November, after which a declining trend took place from December to April (Fig. 4C). This analysis revealed that the transcription of *PsnAP1* remained continuous and stable during the floral primordia formation and floral organ differentiation periods, but the transcription of *PsnAP1s* declined in the inflorescence meristem growth period.

### Constitutive expression of PsnAP1-1 and PsnAP1-2 genes and flowering in transgenic tobacco

**Figure 5:**
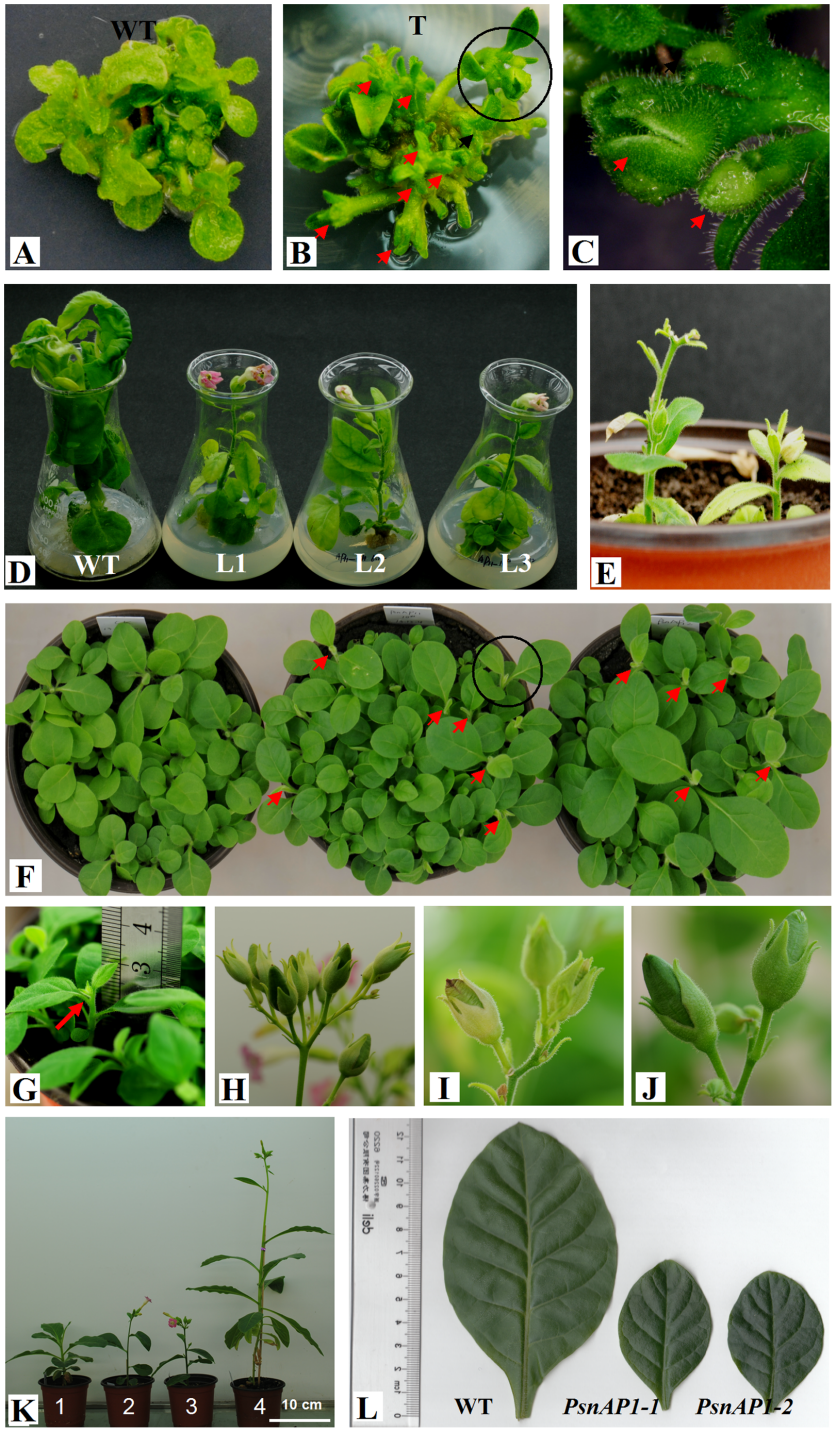
Flowering effect of *PsnAP1-1* and *PsnAP1-2* overexpression in transgenic tobacco. (**A**, **B**) Wild-type (**A**) and transgenic (**B**) leaf explants were cultured on differentiation media for 30 d under normal light conditions. There were many flower buds on the transgenic cultured seedlings. However, no flower buds formed in wild-type plants. (**C**) Close-up of the area circle-outlined in image **B**. (**D**) Wild-type and transgenic explant shoots were cultured on rooting medium for 14 d under normal light conditions. The flowers opened completely on the transgenic plants, WT wild-type, L1-3 three lines of *35S::PsnAP1-1* tobacco. (**E**) The flowers of some transgenic lines faded as soon as the petals opened, and they were unable to form fruits. (**F**) Flower buds were first visible about 4 weeks after sowing. From left to right, the images show wild-type, *35::PsnAP1-1* and *35::PsnAP1-2* plants. (**G**) Close-up view of the area circle-outlined in image **F**. (**H**, **I**, **J**) Fruit phenotype of wild-type (**H**), *35S::PsnAP1-1* (**I**), and *35S::PsnAP1-2* (**J**) plants. (**K**) Phenotype of wild-type and transgenic seedlings at different times: (1) 45-day-old wild-type, (2) 45-day-old *35S::PsnAP1-1*, (3) 45-day-old *35S::PsnAP1-2*, (4) 70-day-old wild-type. (**L**) Second leaf from the top of 45-day-old wild-type, *35S::PsnAP1-1* and *35S::PsnAP1-2* tobacco seedlings.

**Figure 6:**
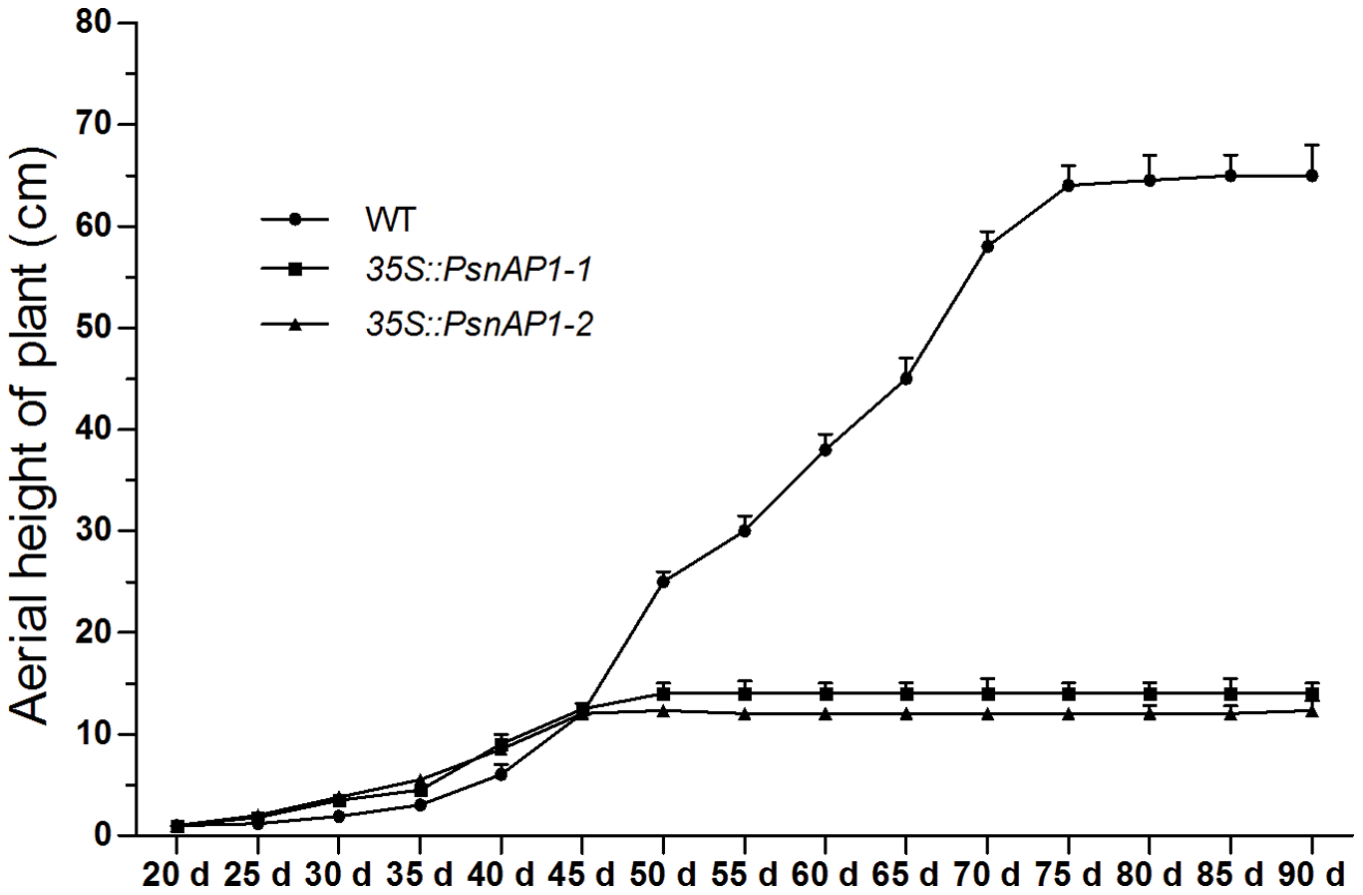
Plant height at different times. The tobacco seedlings were grown in a greenhouse. Each test was performed with 9 seedlings from 3 homozygous transgenic lines.

We first overexpressed *PsnAP1-1* and *PsnAP1-2* genes independently in tobacco under the control of a CaMV 35S promoter (Fig. 4A, B). More than 10 independent transgenic tobacco lines (*35S::PsnAP1*) were generated from each construct, and at least two thirds of them showed clear phenotypic alterations. Northern blot analysis showed that five of the transgenic lines displayed a distinct band of the transgene and that the wild-type line did not, confirming successful transformation and expression of the transgene (Fig. 4C, D). When cultured on differentiation medium, transgenic leaf disks exhibited strikingly different growth morphology, showing that all the normal apical and lateral shoots developed as terminal rudimentary flower in which the petals, stamens, and some floral organs were not well developed, but no flower buds were observed in the wild-type plants (Fig. 5A, B, C). When the shoot explants were cultured on rooting medium, the first flower buds were detected on the transgenic shoots after 7 d, and the flowers opened completely about two weeks later, showing normal flower size and morphology (Fig. 5D). When the cultured plantlets were transferred into soil, the flowers of some transgenic lines faded as soon as the petals opened, and they were unable to form fruits (Fig. 5E). Flower buds were first visible about 4 weeks after sowing. The height of the aerial parts remained under 3 cm (Fig. 5F, G). However, there was no significant morphologic variation in flower and fruit between transgenic and wild-type plants, except that transgenic plants produced fewer fruits (Fig. 5H, I, J). Under long-day conditions with a 16 h light/8 h dark cycle, all the transformants flowered less than 40 d after sowing, whereas wild-type remained for more than 65 d before flowering (Fig. 5K). The transformants were dwarfed, producing less than 8 leaves. Wild-type plants produced more than 15 leaves before flowering. The transformants had much smaller leaves than wild-type tobacco (Fig. 5L). Stem initiation took place much earlier in transgenic plants than that in wild type. However, the transformants were dwarfed, with final aerial heights less than 15 cm, and wild-type plants had final aerial heights more than 60 cm after more than 90 d of growth (Fig. 6). Transgenic plants flowered considerably earlier than wild-type plants, indicating that overexpression of *PsnAP1-1* or *PsnAP1-2* is sufficient to promote the transition from vegetative to reproductive development, suggesting that *PsnAP1-1* and *PsnAP1-2* may be functionally redundant.

### Chlorophyll content and photosynthesis analysis

**Figure 7:**
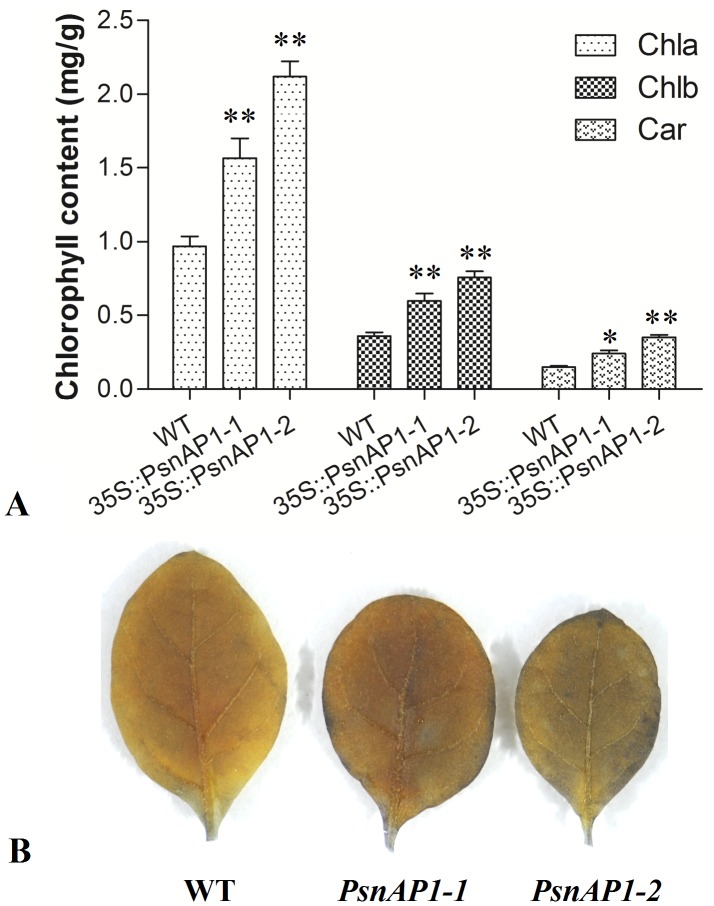
Chlorophyll content and photosynthesis. (**A**) Chlorophyll content. The leaves were collect from 30-day-old tobacco plant. Each transgenic line showed significant differences compared with wild-type by SPSS 11.5 analysis (Student's *t*-test, *P*≤0.01). Values are expressed as means (n = 3 leaves for each test), error bars denote SD. ** *P*≤0.01 for *t* test. WT wild-type, *35S::PsnAP1-1* and *35S::PsnAP1-2* the different transgenic lines. Chla chlorophyll a, Chlb chlorophyll b, Car carotenoid. **B** Starch-iodine staining analysis. After 3 h culture under normal light condition, the leaves were removed for starch-iodine staining analysis. WT wild-type, *PsnAP1-1 35S::PsnAP1-1* tobacco, *PsnAP1-2 35S::PsnAP1-2* tobacco.

The leaves of transgenic plants were darker green than those of wild type. Because chlorophyll gives leaves their green color, the difference of chlorophyll content in wild-type and transgenic plants was compared. Results showed significant differences in the total chlorophyll content (chlorophyll a and b content) and the carotenoid content between young transgenic and young wild-type plants (Fig. 7A). The transgenic leaves also exhibited stronger starch-iodine staining than wild-type leaves (Fig. 7B), indicating higher photosynthesis rate in these transgenics than in the wild type.

### Variations in morphology and development of transgenic tobacco

**Figure 8:**
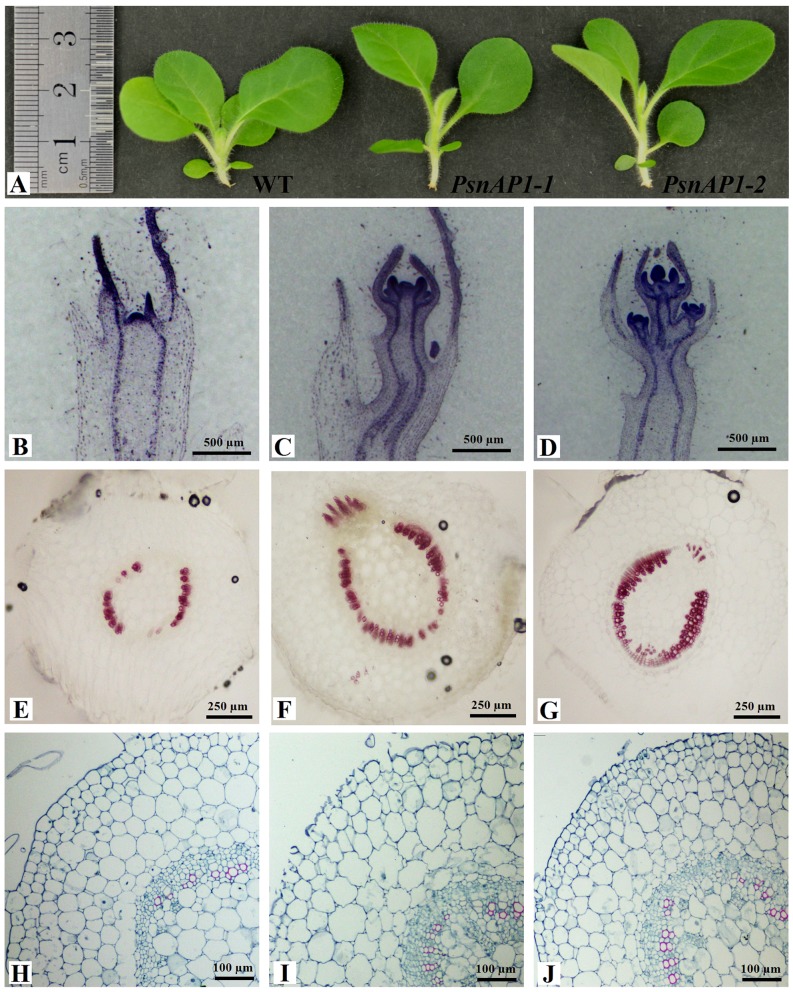
Microscopic observation of tobacco. (**A**) The seedlings from 25-day-old wild-type and transgenic tobacco plants. (**B**, **C**, **D**) The histological observation of apical shoots from 25-day-old wild-type (**B**), *35S::PsnAP1-1* (**C**), and *35S::PsnAP1-2* (**D**) tobacco seedlings. (**E**, **F**, **G**) Hand-cut sections of 25-day-old wild-type (**E**), *35S::PsnAP1-1* (**F**), and *35S::PsnAP1-2* (**G**) tobacco stems were treated with phloroglucinol-HCl for lignin staining. (**H**, **I**, **J**) Histological observation of 25-day-old wild-type (**H**), *35S::PsnAP1-1* (**I**), and *35S::PsnAP1-2* (**J**) tobacco stems were treated with Safranin T and toluidine blue staining. WT wild-type, *PsnAP1-1 35S::PsnAP1-1* line, *PsnAP1-2 35S::PsnAP1-2* line.

In transgenic lines, pleiotropic phenotypes other than early flowering induction were also induced by the overexpression of *PsnAP1-1* and *PsnAP1-2*. As shown in Fig. 8A, stem initiation took place much earlier in transgenic plants (25 d seedlings) than that in wild type. Histological analysis confirmed that flower transition occurred earlier in the shoot apical meristem of transgenic tobacco than that in wild type. The shoot apex of control plants was still in vegetative stage 25 d after germination under long-day conditions, and no inflorescence meristems were present (Fig. 8B), whereas, in transgenic plants, the inflorescence meristem was already formed at this stage (Fig. 8C, D). We next used phloroglucinol-HCl staining to estimate lignification, as phloroglucinol-HCl reacts with coniferaldehyde groups in lignin, and the area and color intensity roughly reflects total lignin content [21]. The stems of transgenic tobacco plants were much more intensely stained than those of wild-type plants (Fig. 8E, F, G). Cell wall lignification is a complex process. It occurs exclusively in higher plants. Its main function is to strengthen the plant vascular body. Safranine T was also used to stain plant tissue sections for lignification of the cell walls. The results of Safranine T staining also indicated that the stems of transgenic tobacco plants were slightly more intensely stained than those of wild type (Fig. 8H, I, J). These staining results suggested that overexpression of *PsnAP1-1* and *PsnAP1-2* may accelerate lignification of cell wall in transgenics.

### Induction of flowering-related genes

**Figure 9:**
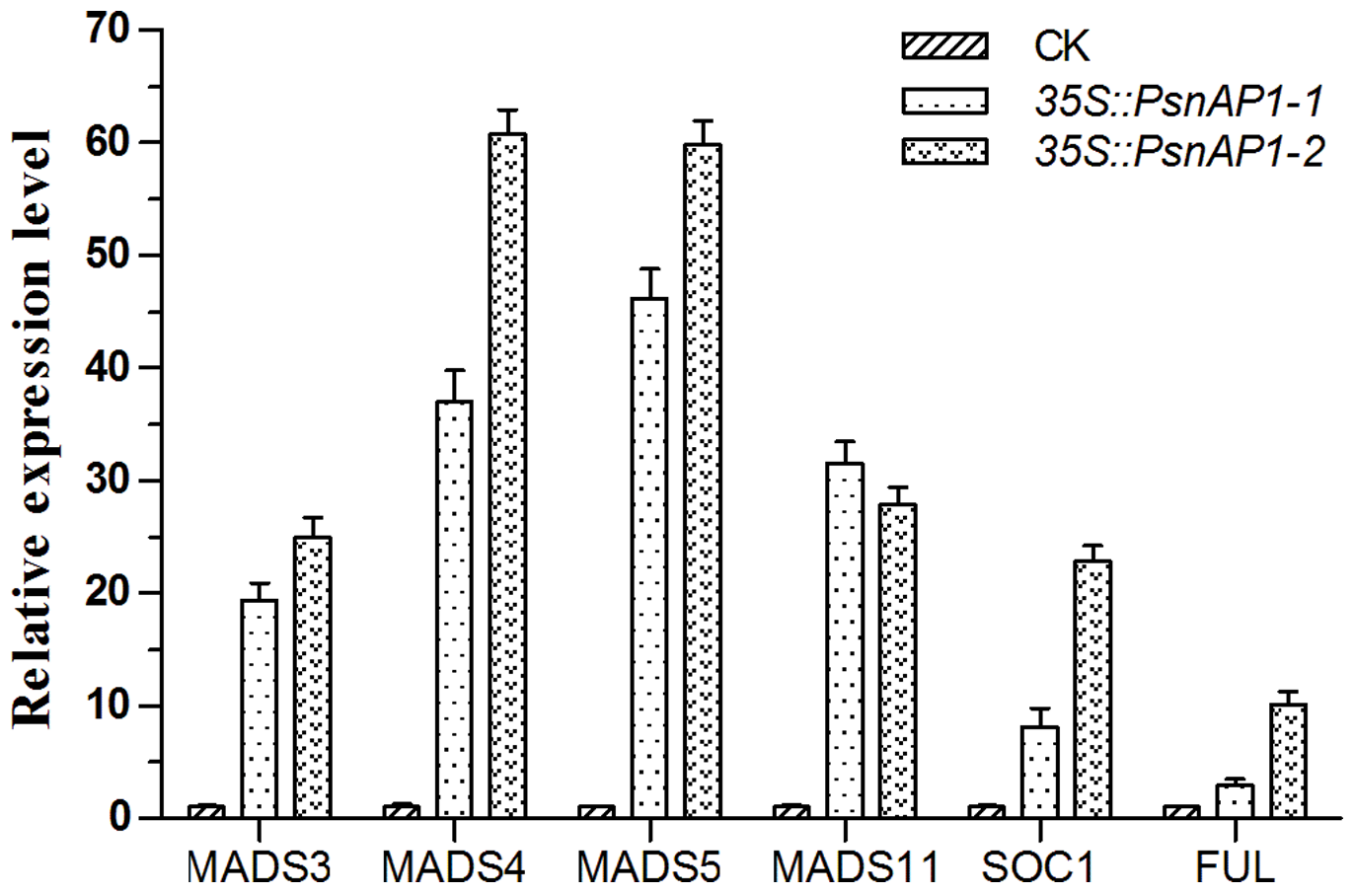
Relative expression levels of flowering-related genes in tobacco. The *35S::PsnAP1-1* and *35S::PsnAP1-2* plants showed significant differences in relative expression level of flowering-related genes compared with wild-type using student's t-test, (*P*≤0.01) by SPSS 11.5 analysis. Values are expressed as mean (n = 3); error bars denote SD. MADS3 *NsMADS3* (No. AF068722), MADS4 *NtMADS4* (No. AF068723), MADS5 *NtMADS5* (No. AF068724), MADS11 *NtMADS11* (No. AF385746), SOC1 *NtSOC1* (No. X76188), FUL *NtFUL* (No. DQ534202) were induced in transgenic plant.

qRT-PCR was performed to estimate the transcription levels of flowering-related genes using the total RNA from aerial parts of 25-day-old transgenic and wild-type tobacco plants, because overexpression of some MADS-box genes was found to induce early flowering and enhance expression of endogenous flowering-related genes in tobacco [22], [23]. Several genes reported to be related to flowering, such as *NsMADS3* (GenBank Accession No. AF068722), *NtMADS4* (No. AF068723), *NtMADS5* (No. AF068724), *NtMADS11* (No. AF385746), *NtSOC1* (No. X76188), and *NtFUL* (No. DQ534202) were selected for analysis. As shown in Fig. 9, there was significantly higher expression of all these genes in transgenic tobacco than in wild type. Specifically, the expression of *NsMADS3*, *NtMADS4*, *NtMADS5*, and *NtMADS11* was more than 20-fold higher in transgenic tobacco than in wild-type plants. The expression of *NtSOC1* and *NtFUL* was more than 10-fold higher in *35S::PsnAP1-2* tobacco than in wild-type plants.

### Characterization of the phenotypes of 35S::PsnAP1-1 and 35S::PsnAP1-2 in wild-type and ap1 mutant of Arabidopsis

**Figure 10:**
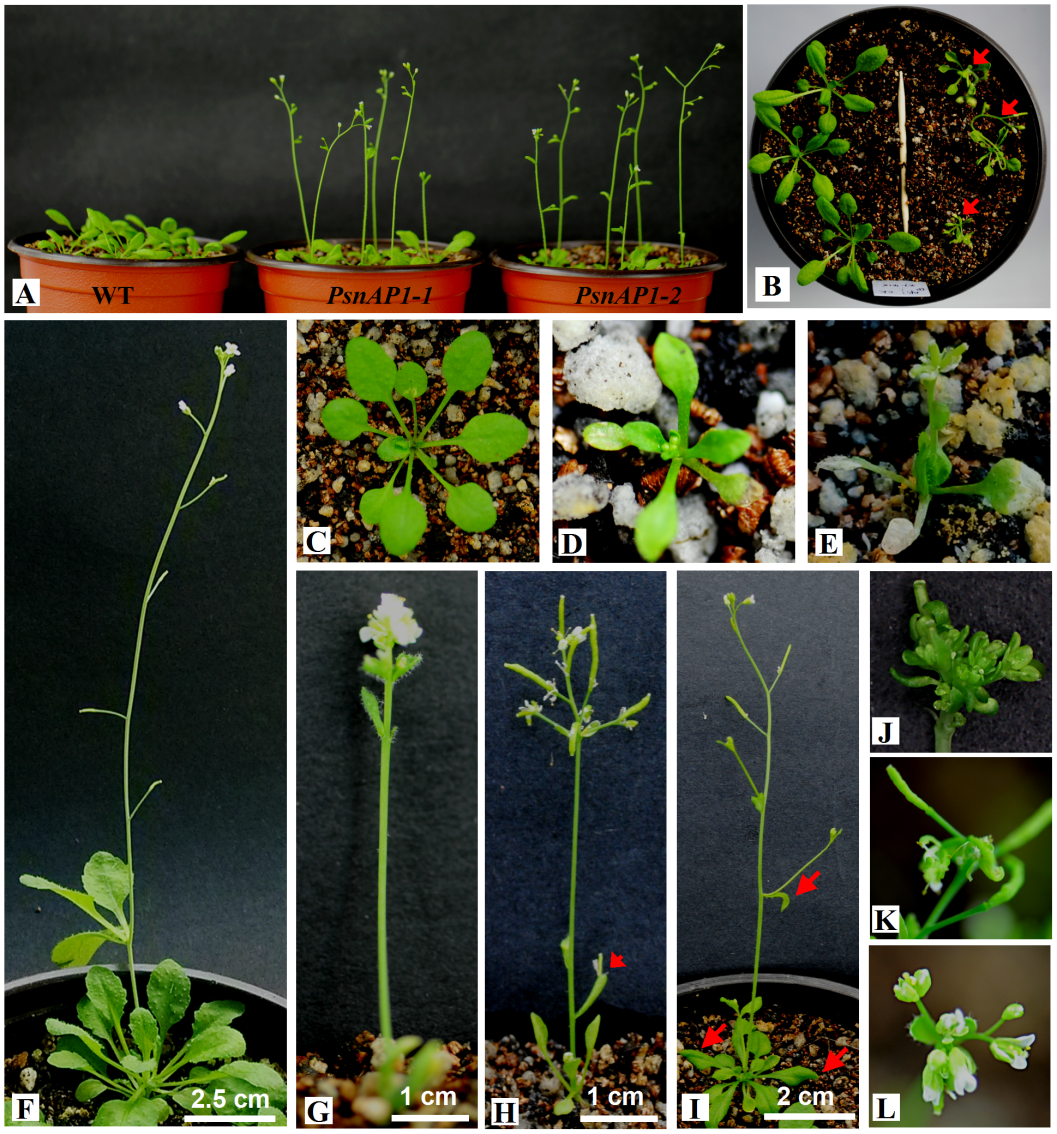
Overexpression of *PsnAP1-1* and *PsnAP1-2* in *Arabidopsis* transgenic lines. (**A**) Comparison of a wild-type Columbia, *35S::PsnAP1-1*, and *35S::PsnAP1-2* 20 d after planting under short-day condition. (**B**) Comparison of a wild-type Columbia and *35S::PsnAP1-1* at about 20 d after planting under long-day conditions, left wild-type, right *35S::PsnAP1-1* transgenic plants, the arrows represent the flowering seedlings. (**C**) Close-up of wild-type 20 d after planting under short-day conditions. (**D**) One *35S::PsnAP1-1* T1 transgenic seedling flowered too early for the seeds to be harvested. (**E**) One *35S::PsnAP1-2* T1 transgenic seedling flowered too early for the seeds to be harvested. (**F**) Wild-type 45 d after planting under short-day conditions. (**G**) A *35S::PsnAP1-2* line with the formation of terminal flowers. (**H**) A *35S::PsnAP1-2* line with deformed flowers. (**I**) A *35S::PsnAP1-1* line with curling leaves and inflorescence branches converted to solitary flowers. (**J, K, L**) Close-up of terminal flowers (**J**), deformed flowers (**K**), and apical and lateral shoots developing as flowers (**L**) in *35S::PsnAP1* lines. WT wild-type, *PsnAP1-1 35S::PsnAP1-1* line in wild-type, *PsnAP1-2 35S::PsnAP1-2* line in wild-type. The arrows represent the locations of observation.

We next overexpressed the *PsnAP1-1* and *PsnAP1-2* in *Arabidopsis* as well as in *ap1* mutant. The constitutive expression of *PsnAP1-1* and *PsnAP1-2* in the wild-type and *ap1-10* mutant plants was verified using Northern blot analysis (Fig. 4E, F). The results showed that overexpression of *PsnAP1-1* and *PsnAP1-2* reduced the vegetative phase of the life cycle of the wild type. Under long-day growth conditions, flowers were first visible on most transgenic lines after approximately 10 d, however, the wild-type plants flowered after an average of approximately 25 d (Table 1, Fig. 10A, B, C). In addition, few T1 transgenic seedlings flowered very early so that they were too fragile to harvest seeds (Fig. 10D, E). However, these T1 transformants were dwarfed, with final aerial heights less than 5 cm, and wild-type showed final aerial height more than 30 cm after more than 60 d of growth (Fig. 10F, G, H, I). A few transgenic lines showed fused terminal flowers, curled leaves, deformed flowers, and lateral shoots that developed into solitary flowers (Fig. 10G–L).

**Table 1:** Flowering time of *PsnAP1-1* and *PsnAP1-2* transgenic *A. thaliana* plants under long-day conditions.

Plant genotypes^1^	Test NO.	Rosette Leaf No. to visible flower bud	Days to visible flower	Plant height/cm
Wild type (Col)	24	8.50±0.53	25.38±0.50	32.90±1.39
*35S::PsnAP1-1* (WT)				
Class I	23	4.00±0.00**	8.8±0.60**	12.19±4.28**
Class II	20	9.27±0.12	28.16±0.92	39.30±2.95
*35S::PsnAP1-2* (WT)				
Class I	20	4.00±0.00**	8.70±1.00**	11.39±0.70**
Class II	18	7.38±0.73	25.07±2.37	28.16±1.96
*ap1-10* (Col)	24	8.02±0.57	26.19±1.32	33.17±1.04
*35S::PsnAP1-1* (*ap1-10*)				
Class I	17	4.00±0.00**	8.55±1.20**	12.10±0.50**
Class II	12	8.55±0.65	22.00±2.10	28.18±3.28
*35S::PsnAP1-2 (ap1-10)*				
Class I	19	4.00±0.00**	8.60±0.40**	10.39±0.35**
Class II	12	7.89±1.15	24.85±2.09	32.44±1.88

1 Plants were classified as Classes I and II based on phenotype imparted by *35S::PsnAP1-1* and *35S::PsnAP1-2* plants.

Plants were kept at 22°C on a 16 h light/8 h dark cycle. Wild-type and *ap1-10* were in Columbia (Col) background.

**Indicates that values are significantly different from control phenotype using student's *t* test (p≤0.01).

**Figure 11:**
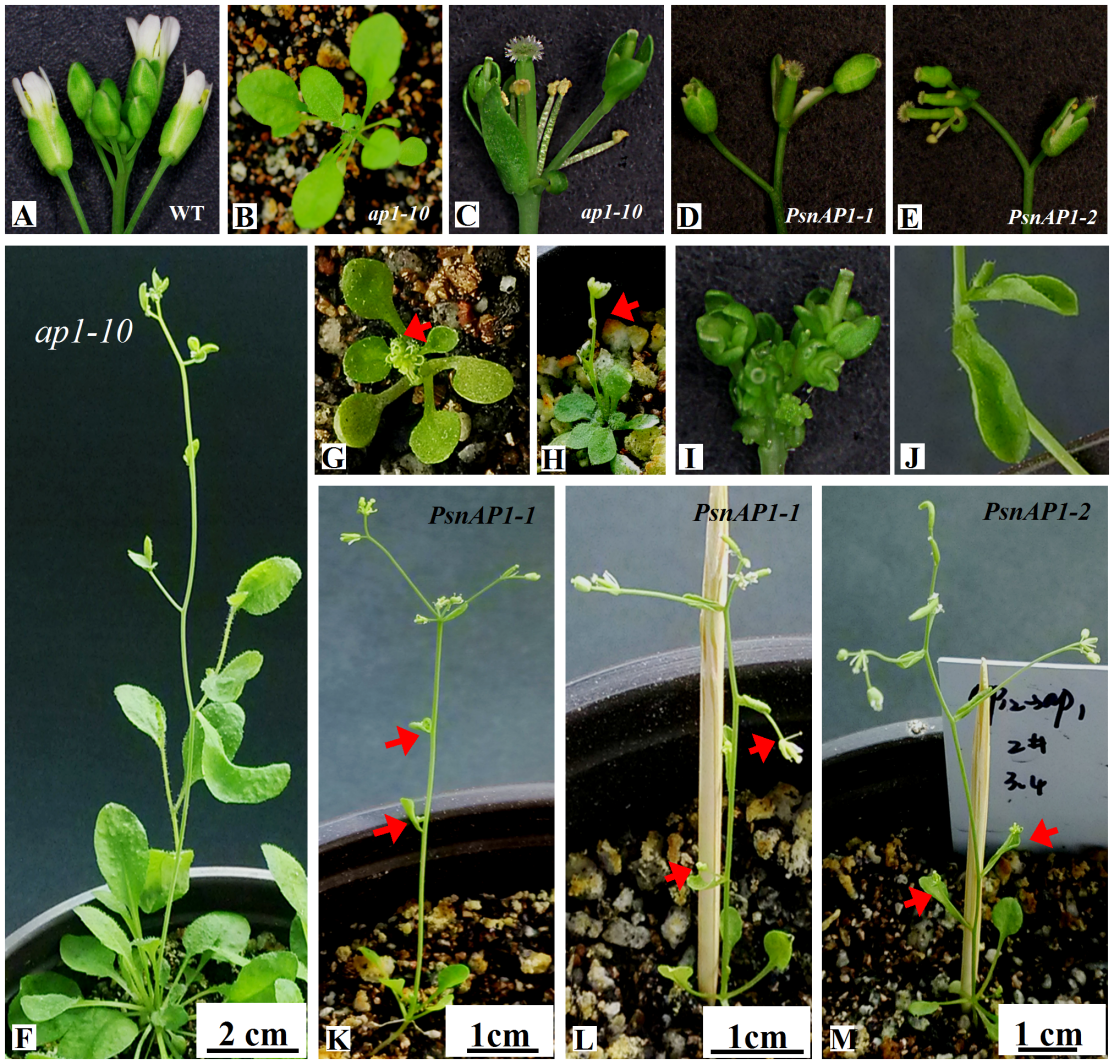
Overexpression of *PsnAP1-1* and *PsnAP1-2* in *ap1-10* mutant *Arabidopsis* caused early flowering. (**A**) The flowers of wild-type Columbia. (**B**) Seedlings of *ap1-10* mutant 20 d after planting under short-day conditions. (**C**) The flowers of *ap1-10* mutant. (**D**) Flowers of representative *35S::PsnAP1-1* in *ap1-10* plants. (**E**) The flowers of representative *35S::PsnAP1-2* in *ap1-10* plant. (**F**) Control *ap1-10* at 45 d after planting under short-day conditions. (**G**) A *35S::PsnAP1-1* transgenic line in *ap1-10* flowered under short-day growth conditions. (**H**) A *35S::PsnAP1-2* transgenic line in *ap1-10* flowered under short-day growth conditions. (**I**, **J**) A *35S::PsnAP1-2* line in *ap1-10* showing formation of terminal flowers (**I**) and curled leaves (**J**). (**K**, **L**) Two *35S::PsnAP1-1* lines in *ap1-10* showed curled bracts subtending the solitary flowers. (**M**) A *35S::PsnAP1-2* line with conversion of secondary inflorescence branches to solitary flowers. WT wild-type, *PsnAP1-1 35S::PsnAP1-1* line in *ap1-10* mutant, *PsnAP1-2 35S::PsnAP1-2* line in *ap1-10* mutant. The arrows represent the locations of observation.

To further test the function of *PsnAP1-1* and *PsnAP1-2*, both ORF sequences, which were under a control of a 35S promoter, were introduced into *Arabidopsis ap1-10* mutant plants. In the Columbia background, *ap1-10* is a strong allele that causes sepals to convert to bract-like structures. Analysis of T3 lines showed that the *ap1-10* plants overexpressed with the *PsnAP1-1* or *PsnAP1-2* were morphologically distinguishable from control plants. However, overexpression of *PsnAP1* didn’t complement the *ap1-10* phenotype (Fig. 11C–E). The *35S::PsnAP1 ap1-10* plants had reduced height, exhibited less branching, and terminated growth prematurely, producing one *ap1-10* like flower each (Fig. 11F, K–M). Flowering time and the number of rosette leaves number were significantly less in the transgenic *ap1-10* plants than those in non-transgenic *ap1-10* plants (Table 1). Under short-day growth conditions, *ap1-10* plants overexpressed with the *PsnAP1-1* or *PsnAP1-2* also flowered after an average of 10 d, but no visible inflorescence shoots were detected on the original *ap1-10* mutants (Fig. 11B, G, H). A few transgenic lines also showed fused terminal flower, curled leaves, and solitary flowers (Fig. 11I, G, K–M).

## Discussion

Genetic and molecular studies in *Arabidopsis* have led to the identification of over 80 genes involved in the transition from vegetative to reproductive growth, providing a base knowledge for the understanding of floral gene interactions [24]. However, the detailed molecular mechanisms controlling these processes in woody perennials are still poorly understood due to difficulties in genetic analysis and transgenic approaches in many of these species. In herbaceous plants, two of these genes, *AP1* and *LFY*, were identified because of their clear phenotype of disrupted SAM productions [25]. Here we identified two *AP1* homologs from *P. simonii* × *P. nigra*, *PsnAP1-1* and *PsnAP1-2*, that belong to MADS-box genes and share 71% and 67% protein sequence identity with their *Arabidopsis* counterpart. *PsnAP1* was found to be a two-copy gene, as reported in other angiosperm [26]. However, neither PsnAP1-1 nor PsnAP1-2 had the prenylation motif “CFAA” at the C-terminus, as found in *Arabidopsis* AP1/SQUA [27], [28]. This motif plays an important role in the determination of the function and specificity of *AP1* in *Arabidopsis* [28]. Instead of the prenylation motif “CFAA,” PsnAP1-1 and PsnAP1-2 have the C-terminal amino acid motif “CFGT” and “GYGA” (Fig. 1). The motif “GYGA” is also found in an AP1 (SdAP1) in *Salix discolor* [26]. The C-terminal amino acid motif “GYGA” is, so far, found only in *Salix* and *Populus*, which are dioecious and have flowers devoid of sepals and petals. An AP1 (VvAP1) from *Vitis vinifera* and the PsnAP1-1 from poplars have a similar “CFT(G)T” motif instead of “CFAA.” ZmAP1 from *Zea mays* and StAP1 from *Solanum tuberosum* share a similar amino acid sequence “HLNA(G)” instead of “CFAA.” These species specific motifs in C-terminus may have played an important role in the functional diversification of the MADS-box genes [29].

The expression profiles of *PsnAP1-1* and *PsnAP1-2* are not similar to those of *AP1* in *Arabidopsis*, in which *AP1* is expressed in young flower primordia but not in the inflorescence meristems [11]. The expression levels of *PsnAP1-1* and *PsnAP1-2* in various tissues and during different developmental periods of the male reproductive buds of poplars indicates that these two genes are involved in the formation of flower primordia, inflorescence meristems, and floral meristems. These expression patterns have also been observed in other woody perennials, such as grapevines and apple, willow, and birch trees [10], [26], [30]–[32].

Our results indicated that the overexpression of *PsnAP1-1* and *PsnAP1-2* significantly promoted the flowering in tobacco, consistent with the similar functions of other *AP1* homologs [6], [30], [32]. In addition to earlier flowering, the transgenic tobacco plants in this study showed accelerated lignification, initiation of axillary bud, and a higher length-to-width ratio in leaves. These developmental changes in the phenotypes of transgenic tobacco may be indication of early flowering.

The main role of chloroplasts is to conduct photosynthesis, where the photosynthetic pigment chlorophyll captures the energy from sunlight and stores it in the energy storage molecules ATP and NADPH while freeing oxygen from water. *35S::PsnAP1-1* and *35S::PsnAP1-2* tobacco plants showed enhanced chloroplast development in leaves. Higher chlorophyll levels were detected in transgenic tobacco, and the transgenic tobacco plants also exhibited much higher rates of photosynthesis than did wild type, as confirmed by the high histochemical staining intensity for starch. Because *PsnAP1-1* and *PsnAP1-2* were not detected in the young poplar leaves, in which the chloroplasts undergo development. The results then suggested that *PsnAP1-1* and *PsnAP1-2* do not play a necessary role in the chloroplast division in poplars. The suggestion is supported by the fact that no significant difference in chloroplast content was observed between *ap1-10* mutants and the wild-type *Arabidopsis*. Therefore, the high chloroplast content in transgenic tobacco plants may be the byproduct of overexpression of *PsnAP1-1* and *PsnAP1-2*.

In this study, we also tested in the transgenic tobacco the expression of endogenous flowering-related genes. We found that the expression of endogenous *NsMADS3*, *NtMADS4*, *NtMADS5*, *NtMADS11*, *NtSOC1*, and *NtFUL* were induced (Fig. 9). Overexpression of *NsMADS2* (ortholog of *NtMADS5*), *NsMADS3*, *NtMADS4*, *Nt*M*ADS11*, *NtSOC1* and *NtFUL* in transgenic tobacco caused early flowering, and ectopic expression of *NtMADS11* was able to rescue the floral organ defects in strong *ap1-1 Arabidopsis* mutant, indicating all these genes play important roles in floral transition in tobacco plants [22], [33], [34]. In present study, aerial parts of 25-day-old tobacco plants were used for qRT-PCR, when the inflorescence meristem was already formed by microscopic observation (Fig. 8C, D). *NsMADS2*, *NsMADS3* and *NtMADS4* were reported to be only present in floral organs [33], [34], and we suppose the enhanced expression levels of *NsMADS2*, *NsMADS3* and *NtMADS4* in transgenic tobacco plants may be attributable to the earlier inflorescence meristem formation caused by overexpression of *PsnAP1-1* and *PsnAP1-2*. *NtSOC1* and *NtFUL* were detectable in vegetative organs in tobacco, however, transcript levels of both genes increased continuously with age of plant and high levels were shown during stage of floral transition [22], so we think that the accelerated reproductive development induced higher expression levels of both genes in transgenic plant than those in wild type. *NtMADS11* was detectable in reproductive organs, and vegetative organs, such as stems and mature leaves, after flowering [34], therefore, we suppose enhanced expression level of *NtMADS11* in transgenic tobacco plants may be attributable to the earlier floral transition.

We also tested whether *PsnAP1-1* and *PsnAP1-2* genes have the similar functions as *AP1* in stimulating flowering in *Arabidopsis* [35]. In transgenic *Arabidopsis*, overexpression of *PsnAP1-1* and *PsnAP1-2* was found to cause markedly early flowering and conversion of inflorescence meristems to flower meristems (Fig. 10). These transgenic lines underwent flowering about 10 d earlier than the controls (Table 1). In addition to early flowering, the *35S::PsnAP1-1* and *35S::PsnAP1-2 Arabidopsis* lines produced flowers of varying phenotypes. Conversion of inflorescence branches to solitary flowers and formation of terminal flowers was observed in these transgenic lines (Fig. 10G, I). The same phenotypes were also observed in transgenic *Arabidopsis* with overexpressed *AP1* homologs from other plant species [26], [33], [36], [37]. Current results demonstrate that *PsnAP1-1* and *PsnAP1-2* were involved in various aspects of flower development in transgenic *Arabidopsis*, suggesting that *PsnAP1-1* and *PsnAP1-2* could act as floral meristem identity genes. In *Arabidopsis*, overexpression of *SdAP1-1* was found to induce the formation of more petals, stamens, and pistils [26]. In our case, a few *35S::PsnAP1-1* and *35S::PsnAP1-2* transgenic *Arabidopsis* lines also formed terminal flowers with many pistils. However, in poplars and willows, neither petals nor sepals would form in their flowers. For this reason, the altered flower phenotype in *Arabidopsis* induced by ectopic expression of *SdAP1* or *PsnAP1* may be due to the abnormal expression of genes related to petal and pistil development, which interacted, directly and indirectly, with *SdAP1* and *PsnAP1*, including *SEP3*, *AP3*, and *PI* [5], [6], [38], [39].

In *Arabidopsis*, *AP1* also specifies the fate of first- and second-whorl floral organs [11]. Complementation of *AP1* function in plants with a strong mutant (*ap1-1*) that develops no petals showed clear restoration of petals [36]. However, unlike that of *Arabidopsis AP1*, overexpression of *PsnAP1-1* or *PsnAP1-2* did not affect *ap1-10* floral morphology to a noticeable extent. *PsnAP1-1* and *PsnAP1-2* may therefore not have retained the role of specifying first- and second-whorl organs during poplar evolution, as in *AP1* homolog of *Antirrhinum majus* [40], [41]. Theissen et al. suggested that the lack of genes with AP1-like function might reflect the more recent evolutionary origin of sepals and petals relative to stamens and carpels [42]. Another possibility is that *PsnAP1-1* and *PsnAP1-2* may not be able to efficiently participate in the gene interactions necessary for floral organ development in *Arabidopsis*. MADS-box gene products are known to form homodimers, heterodimers, and ternary complexes with many other proteins [38], [43]. The 29–33% difference in amino acid sequence between the PsnAP1 and *Arabidopsis* AP1 may reflect the functional divergence between these proteins. In addition, PsnAP1-1 and PsnAP1-2 did not contain the prenylation motif “CFAA,” which plays an important role in the determination of the function and specificity of AP1 in *Arabidopsis* [28]. This current study provides genetic insights of AP1/SQUA functions in dioecious species.

## Supporting Information

Table S1 Primers used in this study.
(XLS)

## References

[1] Borner R, Kampmann G, Chandler J, Gleissner R, Wisman E, et al (2000) A MADS domain gene involved in the transition to flowering in *Arabidopsis*. Plant J. 24: 591–599. 10.1046/j.1365-313x.2000.00906.x 11123798

[2] Kardailsky I, Shukla VK, Ahn JH, Dagenais N, Christensen SK, et al (1999) Activation tagging of the floral inducer FT. Science 286: 1962–1965. 10583961 10.1126/science.286.5446.1962

[3] Bernier G, Périlleux C (2005) A physiological overview of the genetics of flowering time control. Plant Biotechnol J 3: 3–16. 17168895 10.1111/j.1467-7652.2004.00114.x

[4] Mandel MA, Yanofsky MF (1995) A gene triggering flower formation in *Arabidopsis* . Nature 377: 522–524. 7566148 10.1038/377522a0

[5] Bowman JL, Alvarez J, Weigel D, Meyerowitz EM, Smyth DR (1993) Control of flower development in *Arabidopsis thaliana* by *APETALA1* and interacting genes. Development 119: 721–743.

[6] Irish VF, Sussex IM (1990) Function of the *APETALA1* gene during *Arabidopsis* floral development. Plant Cell 2: 741–751. 1983792 10.1105/tpc.2.8.741 PMC159927

[7] Mandel MA, Gustafson-Brown C, Savidge B, Yanofsky MF (1992) Molecular characterization of the *Arabidopsis* floral homeotic gene *APETALA1* . Nature 360: 273–277. 1359429 10.1038/360273a0

[8] Peña L, Martín-Trillo M, Juárez J, Pina JA, Navarro L, et al (2001) Constitutive expression of *Arabidopsis LEAFY* or *APETALA1* genes in citrus reduces their generation time. Nat Biotechnol 19: 263–267. 11231561 10.1038/85719

[9] Kotoda N, Wada M, Kusaba S, Kano-Murakami Y, Masuda T, et al (2002) Overexpression of *MdMADS5*, an *APETALA1*-like gene of apple, causes early flowering in transgenic *Arabidopsis* . Plant Sci 162: 679–687.

[10] Qu GZ, Zheng T, Liu G, Wang W, Zang L, et al (2013) Overexpression of a MADS-Box gene from birch (*Betula platyphylla*) promotes flowering and enhances chloroplast development in transgenic tobacco. PLOS ONE 8: e63398. 23691043 10.1371/journal.pone.0063398 PMC3656909

[11] Huang H, Wang S, Jiang J, Liu G, Li H, et al (2014) Overexpression of *BpAP1* induces early flowering and produces dwarfism in *Betula platyphylla* × *Betula pendula* . Physiol Plant 151: 495–506. 24200078 10.1111/ppl.12123

[12] Zhang B, Su X, Zhou X (2008) A MADS-box gene of *Populus deltoides* expressed during flower development and in vegetative organs. Tree Physiol 28: 929–934. 18381273 10.1093/treephys/28.6.929

[13] Hsu CY, Liu Y, Luthe DS, Yuceer C (2006) Poplar *FT2* shortens the juvenile phase and promotes seasonal flowering. Plant Cell 18: 1846–1861. 16844908 10.1105/tpc.106.041038 PMC1533980

[14] Hsu CY1, Adams JP, Kim H, No K, Ma C, et al (2011) *FLOWERING LOCUS T* duplication coordinates reproductive and vegetative growth in perennial poplar. Proc Natl Acad Sci U S A 108: 10756–10761. 21653885 10.1073/pnas.1104713108 PMC3127867

[15] Tamura K, Dudley J, Ne M, Kumar S (2007) MEGA4: Molecular Evolutionary Genetics Analysis MEGA) software version 4.0. Mol Biol Evol 24: 1596–1599. 17488738 10.1093/molbev/msm092

[16] Niwa Y (2003) A synthetic green fluorescent protein gene for plant biotechnology. Plant Biotechnol 20: 1–11.

[17] Livak K J, Schmittgen TD (2001) Analysis of relative gene expression data using real-time quantitative PCR and the 2−ΔΔCT Method. Methods 25: 402–408. 11846609 10.1006/meth.2001.1262

[18] Chen H, Nelson RS, Sherwood JL (1994) Enhanced recovery of transformants of *Agrobacterium tumifaciens* after freeze-thaw transformation and drug selection. Biotechniques 16 664–668: 670. 8024787

[19] Barnes J D, Balaguer L, Manrique E, Elvira S, Davison AW (1992) A reappraisal of the use of DMSO for the extraction and determination of chlorophylls a and b in lichens and higher plants. Environ Exp Bot 32: 85–100.

[20] Shinano T, Lei TT, Kawamukai T, Inoue MT, Koike T, et al (1996) Dimethylsulfoxide method for the extraction of chlorophylls a and b from the leaves of wheat, field bean, dwarf bamboo, and oak. Photosynthetica 32: 409–415.

[21] Pomar F, Merino F, Ros Barceló A (2002) O-4-Linked coniferyl and sinapyl aldehydes in lignifying cell walls are the main targets of the Wiesner (phloroglucinol-HCl) reaction. Protoplasma 220: 17–28. 12417933 10.1007/s00709-002-0030-y

[22] Smykal P, Gennen J, De Bodt S, Ranganath V, Melzer S (2007) Flowering of strict photoperiodic *Nicotiana* varieties in non-inductive conditions by transgenic approaches. Plant Mol Biol 65: 233–242. 17660946 10.1007/s11103-007-9211-6

[23] Ma G, Ning G, Zhang W, Zhan J, Lv H, et al (2011) Overexpression of *Petunia SOC1*-like gene *FBP21* in tobacco promotes flowering without decreasing flower or fruit quantity. Plant Mol Biol Rep 29: 573–581.

[24] Araki T (2001) Transition from vegetative to reproductive phase. Curr Opin Plant Biol 4: 63–68. 11163170 10.1016/s1369-5266(00)00137-0

[25] Weigel D, Alvarez J, Smyth DR, Yanofsky MF, Meyerowitz EM (1992) *LEAFY* controls floral meristem identity in *Arabidopsis* . Cell 69: 843–859. 1350515 10.1016/0092-8674(92)90295-n

[26] Fernando DD, Zhang S (2006) Constitutive expression of the *SAP1* gene from willow (*Salix discolor*) causes early flowering in *Arabidopsis thaliana* . Dev Genes Evol 216: 19–28. 16228224 10.1007/s00427-005-0026-7

[27] Rodríguez-Concepción M, Yalovsky S, Gruissem W (1999) Protein prenylation in plants: old friends and new targets. Plant Mol Biol 39: 865–870. 10344192 10.1023/a:1006170020836

[28] Yalovsky S, Rodríguez-Concepción M, Bracha K, Toledo-Ortiz G, Gruissen W (2000) Prenylation of the floral transcription factor APETALA1 modulates its function. Plant Cell 12: 1257–1266. 10948247 10.1105/tpc.12.8.1257 PMC149100

[29] Davies B, Schwarz-Sommer Z (1994) Control of floral organ identity by homeotic MADS-box transcription factors. Results and Problems in Cell Differentiation 20: 235–258. 7913550 10.1007/978-3-540-48037-2_11

[30] Sung SK, Yu GH, An G (1999) Characterization of *MdMADS2*, a member of the SQUAMOSA subfamily of genes, in Apple. Plant Physiol 120: 969–978. 10444080 10.1104/pp.120.4.969 PMC59356

[31] Calonje M, Cubas P, Martínez-Zapater JM, Carmona MJ (2004) Floral meristem identity genes are expressed during tendril development in grapevine. Plant Physiol 135: 1491–1501. 15247405 10.1104/pp.104.040832 PMC519065

[32] Elo A, Lemmetyinen J, Turunen ML, Tikka L, Sopanen T (2001) Three MADS-box genes similar to *APETALA1* and *FRUITFULL* from silver birch (*Betula pendula*). Physiol Plant 112: 95–103. 11319020 10.1034/j.1399-3054.2001.1120113.x

[33] Jang S, Hong MY, Chung YY, An G (1999) Ectopic expression of tobacco MADS genes modulates flowering time and plant architecture. Mol Cells 9: 576–586. 10672923

[34] Jang S, An K, Lee S, An G (2002) Characterization of tobacco MADS-box genes involved in floral initiation. Plant Cell Physiol 43: 230–238. 11867703 10.1093/pcp/pcf015

[35] Mandel MA, Yanofsky MF (1995) A gene triggering flower formation in *Arabidopsis* . Nature 377: 522–524. 7566148 10.1038/377522a0

[36] Berbel A, Navarro C, Ferrándiz C, Cañas LA, Madueño F, et al (2001) Analysis of *PEAM4*, the pea *AP1* functional homologue, supports a model for *AP1*-like genes controlling both floral meristem and floral organ identity in different plant species. Plant J 25: 441–451. 11260500 10.1046/j.1365-313x.2001.00974.x

[37] Hsu HF, Huang CH, Chou LT, Yang CH (2003) Ectopic expression of an orchid (*Oncidium Gower Ramsey*) *AGL6*-like gene promotes flowering by activating flowering time genes in *Arabidopsis thaliana* . Plant Cell Physiol 44: 783–794. 12941870 10.1093/pcp/pcg099

[38] Pelaz S, Gustafson-Brown C, Kohalmi SE, Crosby WL, Yanofsky MF (2001) *APETALA1* and *SEPALLATA3* interact to promote flower development. Plant J 26: 385–394. 11439126 10.1046/j.1365-313x.2001.2641042.x

[39] Ng M, Yanofsky MF (2001) Activation of the *Arabidopsis* B class homeotic genes by *APETALA1* . Plant Cell 13: 739–753. 11283333 10.1105/tpc.13.4.739 PMC135542

[40] Bradley D, Ratcliffe O, Vincent C, Carpenter R, Coen E (1993) Inflorescence commitment and architecture in *Arabidopsis* . Science 275: 80–83. 10.1126/science.275.5296.80 8974397

[41] Huijser P, Klein J, Lönnig WE, Meijer H, Saedler H, et al (1992) Bracteomania, an inflorescence anomaly, is caused by the loss of function of the MADS-box gene squamosa in *Antirrhinum majus* . EMBO J 11: 1239–1249. 1563342 10.1002/j.1460-2075.1992.tb05168.x PMC556572

[42] Theissen G, Becker A, Di Rosa A, Kanno A, Kim JT, et al (2000) A short history of MADS-box genes in plants. Plant Mol Biol 42: 115–149. 10688133

[43] Lamb P, McKnight SL (1991) Diversity and specificity in transcriptional regulation: the benefits of heterotypic dimerization. Trends Biochem Sci 16: 417–422. 1776171 10.1016/0968-0004(91)90167-t

